# Aquatic Hyphomycete Taxonomic Relatedness Translates into Lower Genetic Divergence of the Nitrate Reductase Gene

**DOI:** 10.3390/jof7121066

**Published:** 2021-12-11

**Authors:** Joana Mariz, Ricardo Franco-Duarte, Fernanda Cássio, Cláudia Pascoal, Isabel Fernandes

**Affiliations:** 1 Centre of Molecular and Environmental Biology (CBMA), Department of Biology, University of Minho, 4710-057 Braga, Portugal; joanavmariz@gmail.com (J.M.); ricardofilipeduarte@bio.uminho.pt (R.F.-D.); fcassio@bio.uminho.pt (F.C.); cpascoal@bio.uminho.pt (C.P.); 2 Institute of Science and Innovation for Bio-Sustainability (IB-S), University of Minho, 4710-057 Braga, Portugal

**Keywords:** aquatic hyphomycetes, biodiversity, enzymes, functional gene, freshwater ecosystems, leaf litter decomposition

## Abstract

Aquatic hyphomycetes are key microbial decomposers in freshwater that are capable of producing extracellular enzymes targeting complex molecules of leaf litter, thus, being crucial to nutrient cycling in these ecosystems. These fungi are also able to assimilate nutrients (e.g., nitrogen) from stream water, immobilizing these nutrients in the decomposing leaf litter and increasing its nutritional value for higher trophic levels. Evaluating the aquatic hyphomycete functional genetic diversity is, thus, pivotal to understanding the potential impacts of biodiversity loss on nutrient cycling in freshwater. In this work, the inter- and intraspecific taxonomic (ITS1-5.8S-ITS2 region) and functional (nitrate reductase gene) diversity of 40 aquatic hyphomycete strains, belonging to 23 species, was evaluated. A positive correlation was found between the taxonomic and nitrate reductase gene divergences. Interestingly, some cases challenged this trend: *Dactylella cylindrospora* (Orbiliomycetes) and *Thelonectria rubi* (Sordariomycetes), which were phylogenetically identical but highly divergent regarding the nitrate reductase gene; and *Collembolispora barbata (incertae sedis)* and *Tetracladium apiense* (Leotiomycetes), which exhibited moderate taxonomic divergence but no divergence in the nitrate reductase gene. Additionally, *Tricladium chaetocladium* (Leotiomycetes) strains were phylogenetically identical but displayed a degree of nitrate reductase gene divergence above the average for the interspecific level. Overall, both inter- and intraspecific functional diversity were observed among aquatic hyphomycetes.

## 1. Introduction

Small forested streams make up the majority of river basins’ water courses in temperate regions [1]. The main sources of energy and carbon (C) in these ecosystems are allochthonous inputs of non-living organic C, particularly leaf litter from riparian vegetation [2,3]. The decomposition of plant litter is mostly driven by microorganisms and invertebrates [4]. Among microbial decomposers, aquatic fungi have been recognized to play a leading role in this process, exhibiting high biomass and production rates [5,6]. 

Within fungi, a polyphyletic group known as aquatic hyphomycetes stands out as the dominant intermediate in organic matter decomposition and nutrient recycling in headwater stream ecosystems [6,7,8]. Among other features, aquatic hyphomycetes owe their competitive advantage to the ability to produce and secrete a vast array of extracellular degradative enzymes that break down plant cell wall polysaccharides, such as cellulose, hemicellulose, pectin and lignin [9,10].

Nitrogen (N) and phosphorus (P) are known to limit microbial decomposers’ activity, as the demand for these nutrients is usually higher than their availability in streams [1]. Nutrient uptake and assimilation seem to help communities resolve this mismatch [4,11]. In fact, several studies observed a faster decomposition of N- and P-rich organic substrates [12,13,14,15] as well as in N- and P-enriched stream waters [13,15,16].

In freshwater ecosystems, N can be provided from natural (e.g., the atmosphere, soils and vegetation) and anthropogenic (e.g., fertilizers, sewage and atmospheric depositions) sources and occurring in different chemical states, such as ammonium (NH_4_^+^), nitrate (NO_3_^−^) and nitrite (NO_2_) [1,17]. Microbial decomposers are major drivers of N assimilation, usually selecting nitrate as the favoured N source [18,19]. The assimilation process requires two enzymes: nitrate and nitrite reductase. Nitrate reductase is responsible for initiating the metabolic process through the conversion of nitrate to nitrite, and this is necessary for cellular nitrate accumulation in nitrate-assimilating fungi [20,21]. Nitrite reductase mediates the reduction of nitrite to ammonium [20,21]. To the best of our knowledge, only one study showed the presence of nitrate reductase genes in few species of the aquatic hyphomycete genus *Tetracladium* [22].

Several studies observed a positive relationship between aquatic hyphomycete richness and leaf litter decomposition in streams (e.g., [23,24,25]), although considerable functional redundancy among fungi has been recognized [26,27,28]. The role of intraspecific [25] or genetic diversity [28] has received considerably less attention, even with the known relevance of functional traits in maintaining ecosystem processes, particularly under stress [25]. 

Genetic diversity ensures diverse metabolic pathways and might be more relevant than species richness per se for the functioning of microbial communities, particularly under ongoing environmental changes [25,29]. Studies on aquatic fungal genetic diversity have been mostly linked to their taxonomy (e.g., [30,31,32]) with only a limited number of studies addressing functional genetic diversity (e.g., [33,34]). Solé et al. [33] focused on the influence of stream pollution (by metals and xenobiotic organic compounds) on the expression and activity of fungal laccases, which are multicopper oxidase enzymes with distinct functions, including the degradation of lignin compounds. This study found that the aquatic hyphomycete *Clavariopsis aquatica* was able to express five putative laccase genes, including lcc4, which is likely to encode an extracellular laccase potentially involved in lignocellulose breakdown and the detoxification of plant-related phenolics in decaying leaves and woody debris during fungal colonization [33]. 

More recently, the functional diversity of several *Tetracladium* species was assessed, through the detection of carbohydrate active enzymes (e.g., pectate lyase) and secondary metabolites [34]. The results of this study show that *Tetracladium* are well-suited to digest pectate and pectin. Considering the ecological importance of aquatic hyphomycetes for the detrital food-webs in freshwater, knowledge on their functional genetic diversity is crucial to understand the potential impacts of biodiversity loss on nutrient cycling.

The aim of this study was to detect and analyse a functional gene involved in N-cycling from several aquatic hyphomycete species. To that end, we analysed the inter- and intra-specific taxonomic (ITS1-5.8S-ITS2 region) and functional (nitrate reductase gene *euknr*) diversity of 40 aquatic hyphomycete strains belonging to 23 species, using a combination of experimental and in silico approaches. We hypothesized that species with closer taxonomic similarities would exhibit lower functional genetic divergence in the nitrate reductase gene and, consequently, a positive relationship between taxonomic and functional diversity. We expected this divergence to be lower at the intraspecific level than between species (the interspecific level) due to the closer relatedness.

## 2. Materials and Methods

### 2.1. Fungal Strains, Growth Conditions and DNA Extraction

We used a total of 40 strains of aquatic hyphomycetes from 23 species. Sequences of six strains were obtained using in silico approaches (see the section In Silico Assays) and sequences of another four strains were retrieved from Gorfer et al., 2011 [22]. The remaining strains used belonged to the culture collection of the Centre of Molecular and Environmental Biology (CBMA), University of Minho (Table 1). Fungi were grown for 15–30 days in 2% malt extract agar media and kept in the dark at 15 °C. DNA extraction was performed using UltraClean^®^ Soil DNA Isolation Kit (MO BIO Laboratories, Solana Beach, CA, USA) according to the manufacturer’s instructions, except for the final elution step, where 30 µL of Solution S5 were added to increase the final DNA concentration. DNA was stored at −80 °C until used.

### 2.2. Fungal Identification

For morphological identification, agar plugs from the fungal colonies were placed in a 50 mL Erlenmeyer flask with sterile deionised water (renewed weekly) and kept in an orbital shaker (120 rpm) at 15 °C. Every other day, 200 µL of water suspension was collected and placed on a microscope slide. After drying, a drop of 0.1% cotton blue in lactic acid was added to stain the spores for identification under a light microscope [35]. Images were captured using a Leica ICC50 W light microscope (Leica Microsystems, Carnaxide, Portugal) at 400× magnification (Figure S1). 

For cases of lack of sporulation, addition of sterilized alder leaf fragments and/or aeration through air pumps [35] were done (flasks kept at 20 °C). For molecular identification, the ITS1-5.8S-ITS2 region of the rDNA of each fungal strain was amplified by PCR as follows: 12.5 µL of Accuzyme mix (2×) (Bioline, London, UK), 0.4 µM of each primer (ITS1F: 5′-CTTGGTCATTTAGAGGAAGTAA-3′ [36] and ITS4: 5′-TCCTCCGCTTATTGATATGC-3′ [37]) and 2 µL of DNA in a final volume of 25 µL. 

The amplification programme was performed in an iCycler Thermal Cycler (BioRad, Hercules, CA, USA), with an initial denaturation at 94 °C for 2 min, 30 cycles of denaturation (94 °C, 45 s), annealing (56 °C, 45 s) and extension (72 °C, 90 s) and a final extension at 72 °C for 10 min (adapted from [38]). Negative controls with no DNA template were included in each batch. The PCR products were run on a 2% agarose gel with 3% GreenSafe Premium (NZYtech, Lisbon, Portugal) at 80 V for 75 min. The PCR products were purified using Invitrogen’s PureLink^®^ PCR Purification Kit (Invitrogen Life Technologies, Carlsbad, CA, USA) according to the manufacturer’s instructions, with the exception of final elution where 30 µL where used (instead of the recommended 50 µL) to increase the final DNA concentration. 

The DNA concentration was measured using a Nanodrop 1000 Spectrophotometer (Thermo Scientific, Wilmington, DE, USA). DNA sequencing was performed at STABVIDA (Caparica, Portugal) using the primers ITS1F and ITS4. The sequences were trimmed and aligned against each other in order to establish the consensus ITS sequence for each fungal strain and subjected to an individual Basic Local Alignment Search Tool (BLAST; https://BLAST.ncbi.nlm.nih.gov/BLAST.cgi; accessed on 29 November 2019). The parameters for species name attribution were met when ITS sequences presented ≥85% of query coverage and 98–100% of sequence similarity within the results of the BLAST search [39]. The DNA sequences were submitted to Genbank (Table 1).

### 2.3. Nitrate Reductase Gene Amplification

A bibliographic search was made for primers targeting fungal genes potentially involved in nitrogen assimilation. The primers niaD01F/niaD04R and niaD15F/niaD12R successfully amplified the nitrate reductase gene *euknr* (partial sequence) from species of the genus *Tetracladium* [22]. For this reason, these primers were further explored in the present study. Resorting to Sequence Read Archive Nucleotide BLAST (SRABLASTn; https://BLAST.ncbi.nlm.nih.gov/BLAST.cgi; accessed on 3 February 2020), the selected primers were used as queries and located in the genomes of *Articulospora tetracladia* (SRX4652715), *Margaritispora aquatica* (SRX6454163) and *Aquanectria penicillioides* (SRX5023576), the only available aquatic hyphomycete genomes at the date of database accession, to evaluate the primers’ compatibility with other aquatic hyphomycete species. 

As the results displayed a considerable number of hits, these primers were selected to proceed with PCR amplification after optimization. Nested PCR was performed using the two sets of primers: niaD01F: 5′-GTNTGYGCNGGNAA-3′ and niaD04R: 5′-GTNGGRTGYTCRAA-3′ for the first amplification, targeting a wider region and niaD15F: 5′-GGNAAYMGNMGNAARGARCARAA-3′, niaD12R: 5′-AACCANGGRTTRTTCATCATNCC-3′ for a more target-sequence-specific amplification using the products of the first amplification as a DNA template [22]. 

The optimal amplification conditions were as follows: 12.5 µL of Accuzyme mix (2×) (Bioline, London, UK), 2 µM of each primer (niaD01F/niaD04R in the first PCR and niaD15F/niaD12R in the second PCR), 1.25 mM MgCl2, 20 µg/µL BSA, 1.25 µL DMSO (5%) and 2 µL of DNA in a final volume of 25 µL. The cycling conditions were performed as described in Gorfer et al. (2011), using an iCycler Thermal Cycler (BioRad, Hercules, CA, USA). Negative controls with no DNA template were included in each batch. PCR success was assessed by gel electrophoresis (2% agarose gels with 3% GreenSafe Premium (NZYtech, Lisbon, Portugal) run at 80 V for 75 min), considering band(s) with 0.7–1 kb to potentially contain the nitrate reductase gene fragment [22]. PCR products were cleaned using Invitrogen’s PureLink^®^ PCR Purification Kit (Invitrogen Life Technologies, Carlsbad, CA, USA) according to manufacturer’s protocol, and the DNA concentration was measured as above. The DNA was kept at −20 °C.

### 2.4. Cloning and Sequence Alignments

Cloning of the PCR products was performed using NZY-blunt PCR cloning kit (Nzytech, Lisbon, Portugal) according to manufacturer’s protocol. Eight clone colonies were selected from each cloning plate. Plasmids were extracted using NZYMiniprep (Nzytech, Lisbon, Portugal) according to manufacturer’s protocol. For restriction digestion, the purified plasmid DNA was incubated overnight at 37 °C with EcoRI or BamHI as follows: 2 µL of DNA, 1 µL of NZYSpeedyBuffer Colourless (NZYTech, Lisbon, Portugal) and 0.3 µL of Speedy EcoRI/BamHI (NZYTech, Lisbon, Portugal) in a final volume of 10 µL. Enzyme inactivation was performed by incubating at 80 °C for 20 min. 

DNA was run in an agarose gel to confirm the presence of the insert (expected size of approximately 1 kb). Samples presenting bands meeting the expected band sizes were sequenced at STABVIDA (Caparica, Portugal), using the primers T7: 5′-AATACGACTCACTATAG-3′ and U19mer: 5′-TTTTCCCAGTCACGACGT-3′. Sequences were edited using the software BioEdit 7.2.5 [40] and Chromas (http://technelysium.com.au/wp/chromas/; accessed on the 2 March 2020). Forward and reverse sequences from each sample were first trimmed to remove the primers’ binding sites T7 and U19mer and then aligned, with incongruences being manually corrected. Sequences were trimmed again to remove the EcoR V flanking regions. 

After assessing the sequence orientations (due to blunt-end cloning), the last trimming was performed to remove the primers used to perform the PCR amplification (niaD15F and niaD12R). After this, all sequences were subjected to an individual BLASTn search, to evaluate whether they exhibited significant alignments with the targeted functional gene. Finally, multiple alignments were performed for the sequences obtained for each fungal strain in order to create a final consensus sequence.

During this process, some sequences had to be discarded due to: (i) poor quality results in terms of sequencing, (ii) high differences from the other sample sequences for the same isolate, and (iii) not having relevant identity hits in the BLASTn platform sequence(s). The amount of sequences utilized for the final consensus alignments are presented in Table S2. Nitrate reductase partial nucleotide sequences obtained were deposited in GenBank (Accession numbers: MZ812093-109) (Table 1).

### 2.5. In Silico Assays

To the date of this analysis, six aquatic hyphomycete complete genomes were available at NCBI (www.ncbi.nlm.nih.gov/Genomes/; accessed on 30 October 2020), from whole genome shotgun sequencing projects: *Articulospora tetracladia* (NNIBRFG329), *Aquanectria penicillioides* (NNIBRFG19), *Clavariopsis aquatica* (WD(A)-00-1), *Dactylella cylindrospora* (CBS325.70), *Margaritispora aquatica* (NNIBRFG339), and *Thelonectria rubi* (CBS 177.27). Nevertheless, the genomes were not annotated yet. 

We assessed the ITS sequences through primer (ITS1F and ITS4) search for all the six genomes, to confirm identities and establish taxonomic phylogenetic affiliations. The primers niaD15F and niaD12R, as well as a multiple alignment (using the MUSCLE tool from MEGA-X [41]) of our obtained consensus sequences were used to retrieve partial nitrate reductase sequences from all six genomes. Sequence trimming and analysis were performed in BioEdit 7.2.5 [40]. Nucleotide sequences for the ITS1-5.8S-ITS2 region and the nitrate reductase gene (partial sequences) were obtained for all six genomes.

### 2.6. Phylogenetic Analysis

After multiple alignment of the consensus sequences, the sequence divergence was analysed by using p-distance and the phylogenetic trees for the ITS1-5.8S-ITS2 region, and the nitrate reductase gene were generated with neighbour-joining (NJ) method [42] using Maximum composite likelihood distance model using MEGA X [41]. The positions containing alignment gaps and missing data were eliminated in pairwise sequence comparisons. 

Branch support was evaluated using bootstrap analysis (1000 replicates [43]). Sequences from the terrestrial fungus *Tilletiopsis washingtonensis* (D_D11) (Basidiomycota, Exobasidiomycetes) were used as an outgroup to root the ITS1-5.8S-ITS2 (GenBank: HQ115649.1) and nitrate reductase partial nucleotide (GenBank: HQ234861.1) sequence trees. Phylogenetic trees were constructed and the percentage pairwise and overall mean distances were assessed using the same running options.

To estimate the evolutionary relationship between the ITS1-5.8S-ITS2 and the nitrate reductase nucleotide sequences, a Pearson correlation coefficient was calculated [44] using the pairwise distance matrices, considering interspecific variation (species-level average distances) and intraspecific (strain-level individual distances).

## 3. Results

### 3.1. Evolutionary Taxonomic Divergence

**Figure 1:**
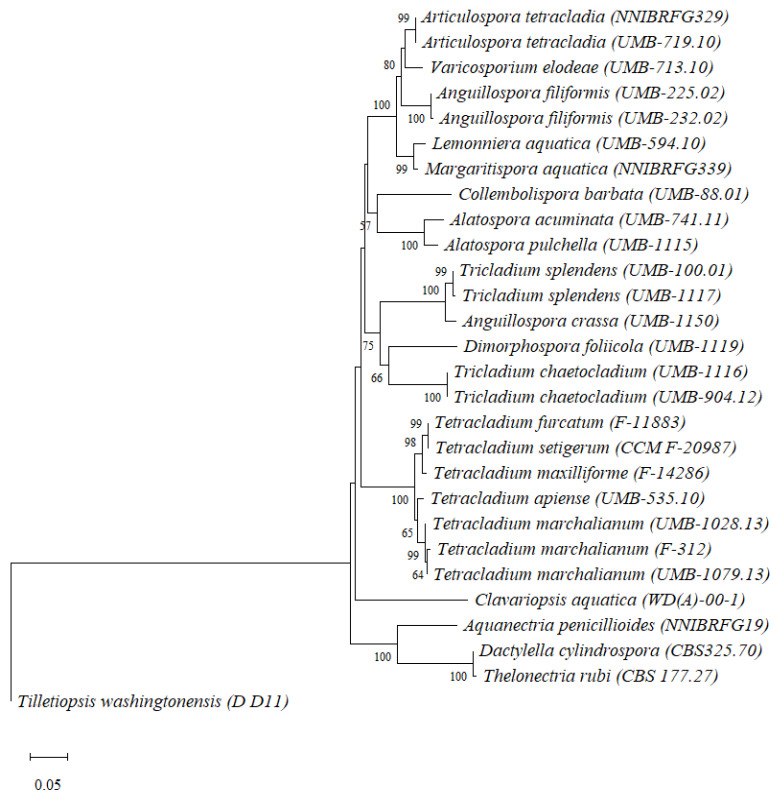
Neighbour joining tree based on ITS1-5.8S-ITS2 nucleotide sequences using Maximum composite likelihood distances. Numbers displayed at the nodes represent bootstrap values above 50%, calculated from 1000 full heuristic replicates. The scale bar represents one base change per 100 nucleotide positions. *Tilletiopsis washingtonensis* (D_D11; Genbank code: HQ115649.1) was used as an outgroup.

Fungal identification of 28 out of the 30 strains was confirmed by both conidial morphology (Figure S1) and ITS sequencing (Table 1). The strains *Alatospora acuminata* UMB-223.02, *Neonectria lugdunensis* UMB-161.01, *Tetracladium marchalianum* UMB-1079.13 and *Tricladium splendens* UMB-100.01 were identified based on molecular information only. The phylogenetic tree for the ITS1-5.8S-ITS2 region of aquatic hyphomycete strains is presented in Figure 1. 

Only species with a positive amplification of nitrate reductase gene (Table 2) were included in this analysis. The evolutionary divergences between aquatic hyphomycete species based on ITS-5.8S-ITS2 sequences ranged between 0 and 26.3 ± 1.8% (Table S3), with an average sequence divergence of 16.6 ± 0.9%. Species from the genus *Tricladium* (*T. chaetocladium* and *T. splendens*; 16.4 ± 1.4% divergence) and *Anguillospora* (*A. filiformis* and *A. crassa*; 16.9 ± 1.6% divergence) exhibited a higher interspecific divergence, grouping in different clades. 

However, this was not the case for the genus *Alatospora* (*A. acuminata* and *A. pulchella*; 4.4 ± 0.9% divergence) and *Tetracladium* (*T. apiense, T. furcatum, T. marchalianum, T. maxilliforme* and *T. setigerum*; 2.2 ± 0.4% divergence), which were grouped within the same clade indicating a lower divergence. The lowest interspecific divergence (0) was found between *Thelonectria rubi* and *Dactyllella cylindrospora*, as well as between *Tetracladium furcatum* and *Tetracladium setigerum*. The highest divergence (26.3 ± 1.8%) was found for the pairs *Clavariopsis aquatica*/*Dimorphospora foliicola* and *Clavariopsis aquatica*/*Thelonectria rubi*. The intraspecific divergences ranged between 0 (*Anguillospora filiformis*, *Tricladium chaetocladium* and *Tricladium splendens*) and 0.2 ± 0.2% (*Articulospora tetracladia* and *Tetracladium marchalianum*) (Table S4).

### 3.2. Evolutionary Divergence of Nitrate Reductase Gene

The amplification success for the nitrate reductase gene was 58.1% (Table 2). Amplification within species exhibited one of three patterns: (i) successful amplifications for all strains (e.g., *Anguillospora filiformis, Tetracladium marchalianum* and *Tricladium chaetocladium*), (ii) variability between isolates, as some strains were successfully amplified and others were not (*Anguillospora crassa, Articulospora tetracladia, Tricladium splendens* and *Varicospium elodeae*) and (iii) no amplification for all strains (e.g., *Neonectria lugdunensis* and *Lunulospora curvula*). 

**Figure 2:**
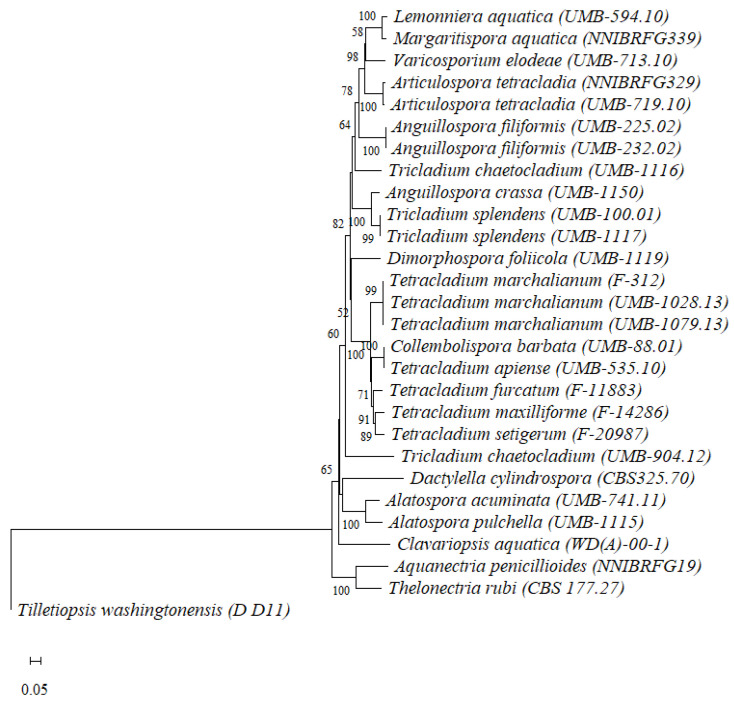
Neighbour joining tree based on nitrate reductase partial nucleotide sequences using Maximum composite likelihood distances. Numbers displayed at the notes represent bootstrap values above 50%, calculated from 1000 full heuristic replicates. The scale bar represents one base change per 100 nucleotide positions. *Tilletiopsis washingtonensis* (D_D11; Genbank code: HQ234861.1) was used as an outgroup.

The eighteen successful amplifications (Table 2) were used for cloning and sequencing. The phylogenetic tree for the nitrate reductase sequences of aquatic hyphomycete strains showed that the evolutionary divergences between aquatic hyphomycete species ranged between 0 and 39.0 ± 1.6% (Figure 2, Table S5) with an average sequence divergence of 26.4 ± 0.9%. 

The species of the genus *Anguillospora* (*A. crassa* and *A. filiformis*), exhibited interspecific differences and were grouped in a separate clade (21.9 ± 1.4% divergence) (Table S5, Figure 2). Species of the genus *Tricladium* (*T. chaetocladium* and *T. splendens*) exhibited even higher evolutionary divergence between their partial nucleotide sequences (26.5 ± 1.2%), also grouping in different clades. Contrarily, species of the genus *Tetracladium* (*T. apiense, T. furcatum, T. marchalianum, T. maxilliforme* and *T. setigerum*) and *Alatospora* (*A. acuminata* and *A. pulchella*) exhibited closer phylogenetic relatedness and grouped in their own clades (divergence of 9.5 ± 0.6% and 13.3 ± 1.2%, respectively; Table S5, Figure 2).

Other species showed a lower divergence: this was the case of *Lemonniera aquatica* and *Margaritispora aquatica* (divergence of 4.8 ± 0.7%) and the strains *Tetracladium apiense* UMB-535.10 and *Collembolispora barbata* UMB-88.01, which shared the same nucleotide sequence (0 sequence divergence). On the contrary, *Aquanectria penicillioides* and *Dactylella cylindrospora* exhibited the highest degree of divergence for the nitrate reductase gene (39.0 ± 1.6%). 

The intraspecific evolutionary divergences ranged between 0 (for strains of *Anguillospora filiformis* and *Tricladium splendens*) and 30.0 ± 1.5% (*Tricladium chaetocladium* strains), with intermediate values for strains of the species *Tetracladium marchalianum* and *Articulospora tetracladia* (Table S4). The highest intraspecific divergence was observed for *Tricladium chaetocladium* strains UMB-904.12 and UMB-1116, which were grouped in different clades (Figure 2).

### 3.3. Relationship between Taxonomic and Functional Evolutionary Divergence

**Figure 3:**
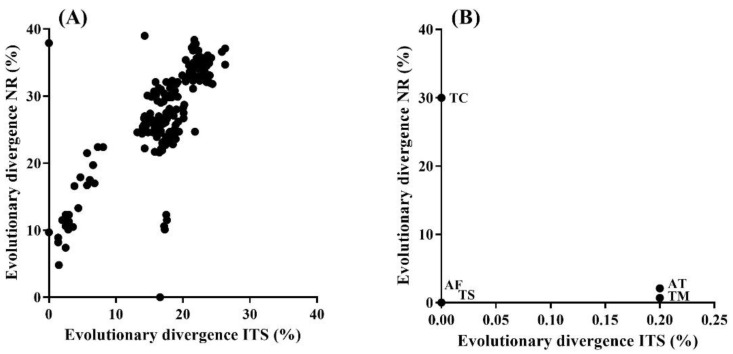
Pearson correlation between evolutionary divergence of ITS1-5.8S-ITS2 (ITS) and nitrate reductase (NR) sequences (**A**) between species (interspecific) and (**B**) within species (intraspecific). Evolutionary divergences were calculated as pairwise distances. AF—*Anguillospora filiformis*, AT—*Articulospora tetracladia*, TM—*Tetracladium marchalianum*, TC—*Tricladium chaetocladium*, and TS—*Tricladium splendens*.

A positive correlation was found between the evolutionary divergence of ITS1-5.8S-ITS2 region and the evolutionary divergence of the nitrate reductase gene of aquatic hyphomycetes species (Pearson-r = 0.77, *p* < 0.0001; Figure 3A). Notwithstanding, evolutionary divergences between some species did not follow this correlation. For instance, *Dactylella cylindrospora* and *Thelonectria rubi* were phylogenetically identical (0 divergence) but exhibited a divergence of 37.9 ± 1.6% for the nitrate reductase gene. On the other hand, *Collembolispora barbata* and *Tetracladium apiense*, which exhibited an evolutionary divergence of 16.6 ± 1.6% for the ITS1-5.8S-ITS2 region, displayed no divergence regarding the nitrate reductase gene.

At the intraspecific level, no correlation was generally found between the evolutionary divergence of ITS1-5.8S-ITS2 region and the evolutionary divergence of the nitrate reductase gene (Pearson-r = −0.36, *p* = 0.553; Figure 3B). The only exceptions showing a positive correlation were *Anguillospora filiformis* and *Tricladium splendens*, but they presented evolutionary divergences of 0 for both ITS1-5.8S-ITS2 region and the nitrate reductase gene. *Articulospora tetracladia* and *Tetracladium marchalianum* exhibited low divergence (<3%) for both ITS1-5.8S-ITS2 region and the nitrate reductase gene. Finally, *Tricladium chaetocladium* showed 0 evolutionary divergence for the ITS1-5.8S-ITS2 region, but a divergence of 30.0 ± 1.5% was found for the nitrate reductase gene.

## 4. Discussion

The present study showed that there is inter- and intraspecific diversity in the nitrate reductase gene of aquatic hyphomycetes. In addition, our data showed a correlation between taxonomic (ITS1-5.8S-ITS2 region) and functional (nitrate reductase gene) divergence, supporting the hypothesis that fungal species that were phylogenetically closer exhibited higher functional gene relatedness. Recently, phylogeny was suggested to play a role in shaping the carbohydrate active enzyme and secondary metabolite content of genomes within *Tetracladium* genus [34]. As phylogenetic relatedness and similarities in functional diversity are expected to be correlated, functional gene analyses can provide new insights on fungal classification and species roles in ecosystem processes [45].

In this study, the ITS1-5.8S-ITS2 phylogenetic tree displayed a clade comprising *Varicosporium elodeae*, *Articulospora tetracladia*, *Anguillospora filiformis*, *Lemonniera aquatica* and *Margaritispora aquatica*, which have been previously observed to be closely related [32]. The results presented in the nitrate reductase phylogenetic tree reflected this close phylogenetic relatedness. We found that ITS1-5.8S-ITS2 and nitrate reductase phylogenetic trees exhibited relevant divergences between species of the genera *Anguillospora* (*A. filiformis* and *A. crassa*) and *Tricladium* (*T. chaetocladium* and *T. splendens*). The polyphyly of the genera *Anguillospora* and *Tricladium* is well documented, comprising anamorphs distributed among Dothideomycetes, Orbiliomycetes and Leotiomycetes [46,47,48]. On the contrary, all *Tetracladium* isolates (*T. apiense*, *T. furcatum*, *T. marchalianum*, *T. maxilliforme* and *T. setigerum*) were comprised in one clade in both ITS1-5.8S-ITS2 and nitrate reductase phylogenetic trees, which is supported by previous studies assessing phylogenetic [32] and nitrate reductase gene diversity [22] in this genus. These results are also sustained by the monophyly proposed for the *Tetracladium* genus (e.g., [49]). A close taxonomic relationship between *Aquanectria penicillioides* and *Thelonectria rubi*, which displayed one of the lowest divergences in ITS1-5.8S-ITS2 (13.78 ± 1.39%) and nitrate reductase gene (22.38 ± 1.37%), was found in our study, and this might be associated with the fact that these are the only species belonging to Hypocreales.

Even though a correlation between phylogeny and functional gene diversity was observed, there were some cases that did not fit this trend. This suggests that close taxonomic phylogenetic relatedness does not inherently translate into highly similar functional gene nucleotide sequences. *Collembolispora barbata* and *Tetracladium apiense* had identical nitrate reductase gene sequences, although they exhibited a high evolutionary divergence of the ITS1-5.8S-ITS2 region. Despite lacking a well-defined class, *Collembolispora barbata* grouped in the same branch of the ITS1-5.8S-ITS2 phylogenetic tree with both *Alatospora* isolates (*A. acuminata* and *A. pulchella*). As *Alatospora* belongs to Leotiomycetes, this close relatedness suggests that this might be the class of *Collembolispora barbata*. However, further studies are necessary in order to confirm this hypothesis. 

On the contrary, *Dactylella cylindrospora* and *Thelonectria rubi* were phylogenetically identical but exhibited a high divergence for the nitrate reductase gene. As *Dactylella cylindrospora* and *Thelonectria rubi* belong to two distinct classes (Orbiliales and Hypocreales, respectively), this degree of phylogenetic similarity was not expected. Gorfer and collaborators [22] found that most nitrate reductase sequences of soil fungi clustered according to their established phylogenies. Notwithstanding, some exceptions were found, like *Doratomyces* sp. (Microascales, Sordariomycetes), which did not cluster with the remaining strains from the Sordariomycetes class in the nitrate reductase gene tree [22].

Surprisingly, we found identical ITS1-5.8S-ITS2 sequences between the two strains of *Tricladium chaetocladium;* however, the degree of divergence for the nitrate reductase gene was above the average for the interspecific level. This indicates the existence of intraspecific functional diversity in aquatic hyphomycetes. Inter- and intraspecific variation was found in the number of carbohydrate binding modules and carbohydrate active enzyme domains between and within strains of the *Tetracladium* genus [34]. Previous studies have found intraspecific functional diversity among aquatic hyphomycetes from differently stressed [50,51,52] and undisturbed [53] environments. Although only two strains of *Tricladium chaetocladium* were used in our study, the results present a proof of concept for the existence of intraspecific genetic variability among aquatic hyphomycete species. Further studies with higher number of strains per species would enhance robustness and infer intraspecific variability in a clearer manner. In particular, it would be relevant to include in future analyses strains isolated from understudied regions like the tropics and the southern hemisphere [54]. This would certainly improve our understanding of the inter- and intraspecific variability of functional genes among aquatic hyphomycetes.

It is relevant to keep in mind that nucleotide sequence divergences do not represent the degree of divergence of their translated amino acid sequences. Due to the redundancy of the genetic code, distinct codons might codify for the same amino acid, meaning that inter- and intraspecific differences in the gene sequences might not necessarily translate into differences in the protein sequences. In order to confirm this, it would be important to retrieve complete nitrate reductase sequences to estimate full amino acid sequences and perform a comparative analysis. 

Furthermore, it is relevant to highlight that the functional genetic information gathered in this study only provides insights for the functional potential of these organisms for the degradation of N compounds. Gene expression analysis would allow for a more accurate prediction of the active metabolism of each organism in ecosystems [55]. Nevertheless, approaches that allow this type of analysis, such as metatranscriptomics, are still very expensive and data-heavy. As there are only a limited number of aquatic hyphomycete genomes available, which are very sparsely annotated, attempting to identify functional gene expression without previous knowledge on the information encoded in that functional gene could constitute a major hardship. That said, the present study represents an important first step towards the understanding of functional diversity, making way for broader, more exciting approaches, such as metatranscriptomics, in investigations to come.

In our study, successful amplification of the nitrate reductase gene was obtained for 58.1% of the fungal strains. However, it is relevant to point out that the lack of amplification does not necessarily mean functional gene absence. Even though the primer pairs utilized were degenerate, they were designed based on terrestrial fungal species [22], which might be distinct from those of aquatic hyphomycetes. Mismatches between the fungal gDNA template and the primers (i.e., all the possibilities comprised by the degenerate primer) could result in a lack of amplification [56], even though the desired nucleotide sequences could still exist in the species genome. These factors must be taken into consideration before making assumptions about the inexistence of a particular functional gene and, consequently, the species functional abilities. Additionally, the design of primers specific to these organisms could potentially increase the amplification success rates but is limited by whole genome sequencing and annotation. 

The recent publication of six aquatic hyphomycete whole genomes (Table 1) in public databases allowed complementing the results obtained in laboratory (with classical molecular techniques) by using in silico approaches to obtain nitrate reductase partial nucleotide sequences. This allowed increasing the diversity of fungal strains pool with the inclusion of new sequences from strains belonging to the classes Orbiliomycetes and Sordariomycetes. The 23 species utilized in our study belong to four classes: Orbiliomycetes, Sordariomycetes, Dothideomycetes and Leotiomycetes (Table 1). Yet, the majority of the strains used in our study (60.9%) belong to Leotiomycetes, which is recognized as one of the most diverse classes of Ascomycota [57]. It would be important to expand this study by covering more species belonging to classes other than Leotiomycetes, in order to understand if the correlation between taxonomic and functional genetic diversity are kept when including species from different classes or even from different phyla (e.g., Basidiomycota). In addition, other early-diverging fungi present in aquatic systems, e.g., Chytridiomycota, can have an important role in organic matter decomposition [58] and thus this correlation should be tested in the future with other fungal groups.

Overall, a correlation between taxonomic and functional gene relatedness has been established for most species. The functional capabilities of each organism do not necessarily correspond to the enzymatic capabilities of a community comprising those organisms. Species interactions within a community are highly variable and context-dependent [59]. Factors, such as temperature, resources availability or stressors, have the ability to alter fungal biodiversity and ecosystem functioning in freshwater ecosystems [16,60,61].Even though the knowledge on fungal diversity and the genetic variation of functional genes is currently expanding, predictions of the potential ecological effects of single organisms combined with abiotic and biotic interactions, community dynamics and ecosystem functioning are still conjectural [62,63]. Establishing links between species’ abilities to cope with environmental change for the maintenance of biogeochemical cycles and ecosystem functioning remains a challenge. Community gene expression analyses, such as metatranscriptomics, have the potential to provide insights into community-level expression [55], which, for aquatic hyphomycetes, would be the first glimpses into their functional gene expression as a community. This approach would allow us to better link functional diversity to biogeochemical processes at an ecosystem scale [64]. Finally, the current study focused on the assimilatory nitrate reductase gene *euknr* [22]. To enable broader conclusions on the aquatic fungal contribution to the biogeochemical nitrogen cycle, the presence and diversity of dissimilatory nitrate reductase genes [65] should also be studied in the future.

## Figures and Tables

**Table 1:** Fungal species and strains utilized in this study. Genome accession numbers correspond to complete genome sequences and GenBank accession numbers comprise the ITS1-5.8S-ITS2 and nitrate reductase sequences of each strain. Information about class and order rows was retrieved from Mycobank (https://www.mycobank.org/; accessed on the 10 September 2021). n/a, not attributed. s/m, Sequence available in the Supplementary Material Table S1.

Species	Culture Collection	Codes	Class	Order	Genbank Accession Number		
					ITS	Nitrate Reductase	Genome
*Alatospora acuminata* Ingold	UMB collection, Portugal	UMB-741.11	Leotiomycetes	Helotiales	MZ773535	MZ812105	n/a
*Alatospora pulchella* Marvanová	UMB collection, Portugal	UMB-1115	Leotiomycetes	Helotiales	MZ773536	MZ812106	n/a
*Anguillospora crassa* Ingold	UMB collection, Portugal	UMB-1150	Dothideomycetes	Pleosporales	MZ77353539	MZ812109	n/a
*Anguillospora filiformis* Greath	UMB collection, Portugal	UMB-225.02	Dothideomycetes	Pleosporales	MZ773533	MZ812102	n/a
		UMB-232.02			OK037621	MZ812103	n/a
*Aquanectria penicillioides* (Ingold) L. Lombard and Crous	UMB collection, Portugal	UMB-304.05	Sordariomycetes	Hypocreales	GQ411325.1	n/a	n/a
	Nakdonggang National Institute of Biological Resources, South Korea	NNIBRFG19			s/m	s/m	PYIU00000000.1
*Articulospora tetracladia* Ingold	UMB collection, Portugal	UMB-72.01	Leotiomycetes	Helotiales	OK605571	n/a	n/a
		UMB-712.10			OK605572	n/a	n/a
		UMB-719.10			OK037616	MZ812096	n/a
		UMB-1144			OK605573	n/a	n/a
	Nakdonggang National Institute of Biological Resources, South Korea	NNIBRFG329			s/m	s/m	GCA_003415645.1
*Clavariopsis aquatica* De Wild.	Leibniz-Institute of Freshwater Ecology and Inland Fisheries, Germany	WD(A)-00-1	Dothideomycetes	Pleosporales	s/m	s/m	GCA_013620735.1
*Collembolispora barbata* Marvanová, Pascoal and Cássio	UMB collection, Portugal	UMB-88.01	n/a	n/a	MZ773532	MZ812101	n/a
*Dactylella cylindrospora* (R.C. Cooke) A. Rubner	Laboratory for Conservation and Utilization of Bio-Resources and Key Laboratory for Microbial Diversity, Southwest China	CBS325.70	Orbiliomycetes	Orbiliales	s/m	s/m	GCA_012184295.1
*Dimorphospora foliicola* Tubaki	UMB collection, Portugal	UMB-1119	Leotiomycetes	Helotiales	MZ773538	MZ812108	n/a
*Neonectria lugdunensis* (Sacc. and Therry) L. Lombard and Crous	UMB collection, Portugal	UMB-3.00	Sordariomycetes	Hypocreales	OK605574	n/a	n/a
		UMB-160.01			OK605575	n/a	n/a
		UMB-161.01			OK605576	n/a	n/a
		UMB-311.06			OK605577	n/a	n/a
*Lemonniera aquatica* De Wild.	UMB collection, Portugal	UMB-594.10	Leotiomycetes	Helotiales	MZ773530	MZ812094	n/a
*Lunulospora curvula* Ingold	UMB collection, Portugal	UMB-108.01	n/a	n/a	OK605578	n/a	n/a
		UMB-498.09			OK605579	n/a	n/a
*Margaritispora aquatica* Ingold	Nakdonggang National Institute of Biological Resources, South Korea	NNIBRFG339	Leotiomycetes	Helotiales	s/m	s/m	GCA_007644065.1
*Tetracladium apiense* R.C. Sinclair and Eicker	UMB collection, Portugal	UMB-535.10	Leotiomycetes	Helotiales	OK037615	MZ812093	n/a
*Tetracladium furcatum* Descals	Biology Dept., Mount Allison University, Sackville, NB, Canada	F-11883	Leotiomycetes	Helotiales	AF411026.1	HQ234857.1	n/a
*Tetracladium marchalianum* De Wild.	UMB collection, Portugal	UMB-1028.13	Leotiomycetes	Helotiales	OK037619	MZ812099	n/a
		UMB-1079.13			OK037620	MZ812100	n/a
	Biology Dept., Mount Allison University, Sackville, NB, Canada	F-312			AF411023.1	HQ234858.1	n/a
*Tetracladium maxilliforme* (Rostr.) Ingold	Biology Dept., Mount Allison University, Sackville, NB, Canada	F-14286	Leotiomycetes	Helotiales	AF411027.1	HQ234859.1	n/a
*Tetracladium setigerum* (Grove) Ingold	Institute for Environmental Sciences, University of Koblenz-Landau, Germany	CCM F-20987	Leotiomycetes	Helotiales	KU519120.1	HQ234860.1	n/a
*Thelonectria rubi* (Osterw.) C. Salgado & P. Chaverri	Agricultural Research Service, United States Department of Agriculture, USA	CBS 177.27	Sordariomycetes	Hypocreales	s/m	s/m	GCA_013420875.1
*Tricladium chaetocladium* Ingold	UMB collection, Portugal	UMB-904.12	Leotiomycetes	Helotiales	OK037617	MZ812097	n/a
		UMB-1116			MZ773531	MZ812095	n/a
*Tricladium splendens* Ingold	UMB collection, Portugal	UMB-100.01	Leotiomycetes	Helotiales	OK037618	MZ812098	n/a
		UMB-414.09			OK605580	n/a	n/a
		UMB-1117			MZ773537	MZ812107	n/a
*Varicosporium elodeae* W. Kegel	UMB collection, Portugal	UMB-310.06	Leotiomycetes	Helotiales	OK605581	n/a	n/a
		UMB-713.10			MZ773534	MZ812104	n/a
		UMB-878.12			OK605582	n/a	n/a

**Table 2:** Amplification success of the nitrate reductase functional gene. Yes, successful amplification; and No, unsuccessful amplification.

Species	Strain Code	Nitrate Reductase Amplification Success
*Alatospora acuminata*	UMB-741.11	Yes
*Alatospora pulchella*	UMB-1115	Yes
*Anguillospora crassa*	UMB-217.02	No
*Anguillospora crassa*	UMB-1150	Yes
*Anguillospora filiformis*	UMB-225.02	Yes
*Anguillospora filiformis*	UMB-232.02	Yes
*Aquanectria penicillioides*	UMB-304.05	No
*Articulospora tetracladia*	UMB-72.01	No
*Articulospora tetracladia*	UMB-712.10	Yes
*Articulospora tetracladia*	UMB-719.10	Yes
*Articulospora tetracladia*	UMB-1144	No
*Collembolispora barbata*	UMB-88.01	Yes
*Dimorphospora foliicola*	UMB-1119	Yes
*Lemonniera aquatica*	UMB-594.10	Yes
*Lunulospora curvula*	UMB-108.01	No
*Lunulospora curvula*	UMB-498.09	No
*Neonectria lugdunensis*	UMB-3.00	No
*Neonectria lugdunensis*	UMB-160.01	No
*Neonectria lugdunensis*	UMB-161.01	No
*Neonectria lugdunensis*	UMB-311.06	No
*Tetracladium apiense*	UMB-535.10	Yes
*Tetracladium marchalianum*	UMB-1028.13	Yes
*Tetracladium marchalianum*	UMB-1079.13	Yes
*Tricladium chaetocladium*	UMB-904.12	Yes
*Tricladium chaetocladium*	UMB-1116	Yes
*Tricladium splendens*	UMB-100.01	Yes
*Tricladium splendens*	UMB-414.09	No
*Tricladium splendens*	UMB-1117	Yes
*Varicosporium elodeae*	UMB-310.06	No
*Varicosporium elodeae*	UMB-713.10	Yes
*Varicosporium elodeae*	UMB-878.12	No
Amplification success rate (%)		58.1

## Data Availability

The data presented in this study are openly available in Genbank and Supplementary material. Genbank accession numbers are listed in Table 1.

## Supplementary Materials

The following are available online at https://www.mdpi.com/article/10.3390/jof7121066/s1, Figure S1: Conidia of aquatic hyphomycetes. Table S1: ITS1-5.8S-ITS2 and Nitrate reductase DNA sequences retrieved from full genomes available in NCBI and referred in Table 1. Table S2: Number of clone colonies used for enzymatic restriction (EcoRI and BamHI), DNA inserts sent for sequencing and sequences utilized in the alignments of each fungal isolate (using all the sequencing efforts; both digestions with EcoRI and BamHI). Table S3: Evolutionary divergence (%) between aquatic hyphomycete species based on ITS-5.8S-ITS2 sequences. Table S4: Evolutionary divergence (%) within aquatic hyphomycete species based on ITS-5.8S-ITS2 and Nitrate reductase sequences. Table S5: Evolutionary divergence (%) between aquatic hyphomycete species based on nitrate reductase partial nucleotide sequences.
