# Supplementary material for: Aquatic Hyphomycete Taxonomic Relatedness Translates into Lower Genetic Divergence of the Nitrate Reductase Gene
Source: J Fungi (Basel). 2021 Dec 11;7(12):1066. doi: 10.3390/jof7121066 (PMC8708292; doi:10.3390/jof7121066)
Supplement: Supplementary file 1 [file jof-07-01066-s001.zip › jof-1470667-supplementary.pdf]

Aquatic Hyphomycete Taxonomic Relatedness Translates into Lower Genetic Divergence of the Nitrate Reductase Gene

Joana Mariz [1,2], Ricardo Franco-Duarte [1,2], Fernanda Cássio[1,2], Cláudia Pascoal[1,2†] and Isabel Fernandes [1,2†]*

[1] Centre of Molecular and Environmental Biology (CBMA), Department of Biology, University of Minho, 4710-057 Braga, Portugal; joanavmariz@gmail.com; ricardofilipeduarte@bio.uminho.pt; fcassio@bio.uminho.pt; cpascoal@bio.uminho.pt

[2] Institute of Science and Innovation for Bio-Sustainability (IB-S), University of Minho, 4710-057 Braga, Portugal

* Correspondence: isabelrodriguesfernandes@bio.uminho.pt;

† Co-last authors

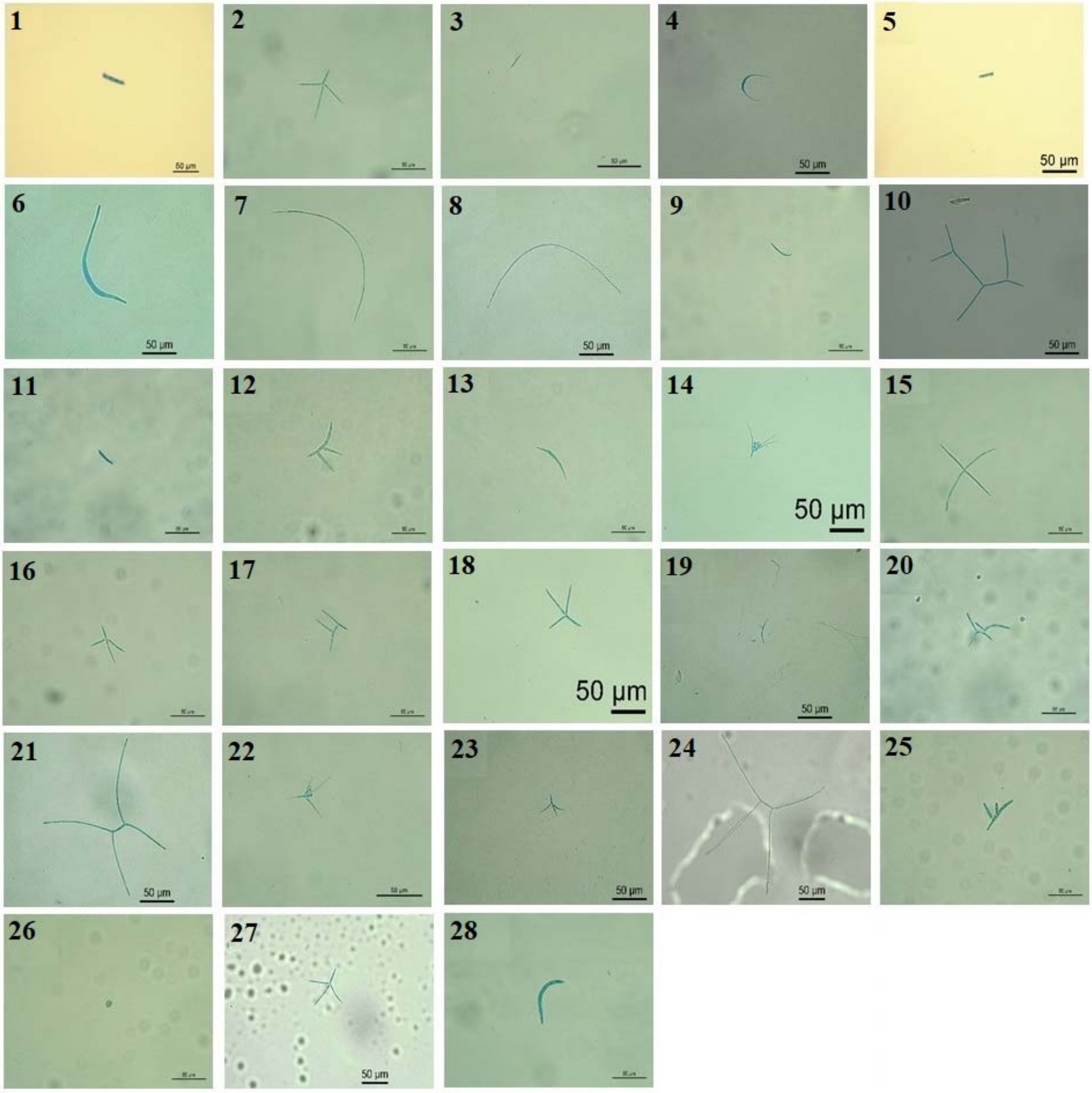

Figure S1. Conidia of aquatic hyphomycetes. 1- *Neonectria lugdunensis* (UMB-3.00), 2- *Articulospora tetracladia* (UMB-72.01), 3- *Collembolispora barbata* (UMB-88.01), 4- *Lunulospora curvula* (UMB-108.01), 5- *Neonectria lugdunensis* (UMB-160.01), 6- *Anguillospora crassa* (UMB-217.02), 7- *Anguillospora filiformis* (UMB-225.02), 8- *Anguillospora filiformis* (UMB-232.02), 9- *Flagellospora penicillioides* (UMB-304.05), 10- *Varicosporium elodeae* (UMB-310.06), 11- *Neonectria*

lugdunensis (UMB-311.06), 12- *Tricladium splendens* (UMB-414.09), 13- *Lunulospora curvula* (UMB-498.09), 14- *Tetracladium apiense* (UMB-535.10), 15- *Lemonniera aquatica* (UMB-594.10), 16- *Articulospora tetracladia* (UMB-712.10), 17- *Varicosporium elodeae* (UMB-713.10), 18- *Articulospora tetracladia* (UMB-719.10), 19- *Alatospora acuminata* (UMB-741.11), 20- *Varicosporium elodeae* (UMB-878.12), 21- *Tricladium chaetocladium* (UMB-904.12), 22- *Tetracladium marchalianum* (UMB-1028.13), 23- *Alatospora pulchella* (UMB-1115), 24- *Tricladium chaetocladium* (UMB-1116), 25- *Tricladium splendens* (UMB-1117), 26- *Dimorphospora foliicola* (UMB-1119), 27- *Articulospora tetracladia* (UMB-1144), 28- *Anguillospora crassa* (UMB-1150).

Table S1. ITS1-5.8S-ITS2 and Nitrate reductase DNA sequences retrieved from full genomes available in NCBI and referred in Table 1.

Species	Genbank accession number	ITS1-5.8S-ITS2 sequence	Nitrate reductase sequence
Articulospora tetracladia	NNIBRFG329	AAGTCGTAACAAGGTTTCCGTAGGT<br>GAACCTGCGGAAGGATCATTACAG<br>AGTTCATGCCCTCACGGGTAGATCT<br>CCCACCCTTGAATATTATACCTTAG<br>TTGCTTTGGCAGGCCGTGGAAACAC<br>CACGGGCTTTGGCTTGTGCGTGCCT<br>GCCAGAGGAAACAAACTCTGTTTTT<br>AGTGATGTCTGAGTACTATATAATA<br>GTTAAAACTTTCAACAACGGATCTC<br>TTGGTTCTGGCATCGATGAAGAACG<br>CAGCGAAATGCGATAAGTAATGTG<br>AATTGCAGAATTCAGTGAATCATCG<br>AATCTTTGAACGCACATTGCGCCCT<br>GTGGTATTCCGCAGGGCATGCCTGT<br>TCGAGCGTCATTTCAACCCATCAAG<br>CTTACGCTTGGTATTGGGGCCTGCG<br>GTTTCGCAGCCTCTAAACTCAGTGG<br>CGGTGCAACTGAGCTCTGAGCGTAG<br>TAATTTTTCTCGCTATAGGGTCTCGG<br>TTGTTACTTGCCAGCAACCCCCAAT<br>TTTTATCAGGTTGACCTCGGATCAG<br>GTAGGGATACCCGCTGAACTTAA	CAGGTCAGAAAGTCGAAGGGATTTT<br>CGTGGGGAGCGGCTGGTGTCTCCAC<br>GGCGCTGTGGACGGGTGTTCCCATA<br>AGCGAGTTGATCAGGAGAGCCGTG<br>CCGATGCGAGGAGCAAAGTATTTCT<br>GTATGGAAGGCGCCGACAAACTTC<br>CTAATGGCTACTATGGCACCAGTAT<br>CAAATTAAACTGGGCAATGGATCC<br>AAACAGGGGCATCATGTTGGCCCA<br>CAAGATGAATGGAGAAACACTCAC<br>ACCAGATCACGGCAAGCCACTTCGT<br>GTTGTTATCCCGGGTCAGATCGGCG<br>GCCGCAGCGTGAAATGGCTGAAGA<br>AGATCATCATTAGTGCAGAGCCAA<br>GCGATAACTGGTATCACATCTACGA<br>CAACAGAGTTCTTCCTACAATGGTG<br>ACTCCTGAGATGGGTGCCGCCGACC<br>CAAGCTGGTGGACAGATGAGCGAT<br>ACGCCATTTACGACTTGAGTACGAA<br>CAGTGCCACTGCCTACCCTGCACAC<br>GACGAGCAGGTATCTCTTGTGGACG<br>GTCCCAAGACATACAAAGTCAGAG<br>GATATGCTTACGGTGGAGGTGGACG<br>AAGAGTCACCCGCGTTGAAGTGAC<br>ACTTGACAAAGGCAAATCATGGGC<br>TCTGGCAAATATCAGATACCACGAG<br>GATGATTACCGAGAGGCAGCGGAA<br>GACGAGATGCTTTACGGCGGCAAG<br>CTCGACATGGGATGGCGAGAGACT<br>TGCTTTGCATGGTGCTTTTGGGATCT<br>CGATCTGAGTGTTGAAGATATGGCT<br>TTGTCTGGCGACATCATGGTTCGAG<br>CAATGGACGAAAGCATGAACGTTC<br>AGCCACGTGATATGTACTGGAGTGT<br>GCTG
Aquanectria penicillioides	NNIBRFG19	AAGTCGTAACAAGGTCTCCGTTGGT<br>GAACCAGCGGAGGGATCATTACCG<br>AGTTTACAACTCCCAAACCCTGTGA<br>ACTATACCATATTGTTGCCTCGGCG<br>GCGTCCTGCTTCACGGCGGGCCCGC<br>CAGAGGACCCAACTCTTGTATTTGA<br>ATTGAGTCTTCTCTGAGTGATACAA<br>GTAATAAATCAAAACTTTCAACAAC<br>GGATCTCTTGGTTCTGGCATCGATG	ATAGTAAGAAAATCAAAAGGGTTT<br>TCCTGGGGCGCCGCCGGTCTTTCAA<br>CAGCATTGTGGACTGGAGTTCCCAT<br>CCACAACCTTTTGGCGGCTGTGCGT<br>CCCAAACGAGGTGCCAGATATGTTT<br>GCTTCGAGGGCTCTGACAAGCTGCC<br>GAATGGCTACTATGGAACGTCTATC<br>AAGTTGAATTGGTGCATGGATCCGA<br>ATAGAGGTGTCATGGTTGCCCACAA

		AAGAACGCAGCGAAATGCGATAAG<br>TAATGTGAATTGCAGAATTCAGTGA<br>ATCATCGAATCTTTGAACGCACATT<br>GCGCCCGCCAGTATTCTGGCGGGCA<br>TGCCTGTTCGAGCGTCATTTCAACC<br>CTCAAGCCCCCCGGGCTTGGTGTTG<br>GAGATCGGCAAAACGGCCCCCTCG<br>GGGTTCGCGCCGTCTCCCAAATCTA<br>GTGGCGGTCTCGCTGTAGCTTCCTCT<br>GCGTAGTAACTCACCTCGCACTGGG<br>ACTGGGCGCGGCCACGCCGTTAAA<br>CACCCCACTTCTGAAGGTTGACCTC<br>GGATCAGGTAGGACTACCCGCTGA<br>ACTTAA	AATGAACGGAGACCCTTTGCATCCC<br>GATCATGGCAAGCCGGTTCGCATAA<br>TAATTCCTGGCCAGATCGGAGGTAG<br>GAGCGTCAAGTGGCTGAAACGGAT<br>CATCGTCACTCCTGGGCCGAGTGAG<br>AATTGGTACCACATCTTCGACAATC<br>GCGTGCTTCCCACCATGATCACTCC<br>TGAGGCTAGCGCAGATCTACCCGA<br>GGTCTGGAGAGACGAGAATTATGC<br>AATCTACGACCTAAATGTCAACAGC<br>GCAATATGCTATCCCAGCCATGATG<br>AAATCGTGCCTCTGGGAAAGAGTG<br>GCATCACTTACACTGCACGAGGATA<br>CGCCTACAGCGGTGGCGGAAGGCG<br>AGTCACAAGGGTTGAAGTCACCCTA<br>GACAAGGGGAAATCTTGGAGACTC<br>GCAAATGTTGATTACCCAGAAGAC<br>GCCTATCGACTTGCGCCGGAAGGG<br>GACACGCTGTTTGGCGGGAAGGTTG<br>ACATGTGGTGGCGGGAGACGTGTTT<br>CTGTTGGTGCTTCTGGGAACTTAAG<br>ATTCCACTAGATGACTTGCGAGATG<br>CTGAAGACATCATGATTCGAGCAAT<br>GGACGAATCGATGATGGTGCAGCC<br>TCGCGATATGTACTGGAGTGTGCTC
Clavariopsis aquatica	*WD(A)-00-1*	AAGTCGTAACAAGGTTTCCGTAGGT<br>GAACCTGCGGAAGGATCATTACCGT<br>GGGGATTCGTCCCCATTGAGATAGC<br>ACCCTTTGTTTATGAGTACCCTGTTT<br>CCTCGGCGGGCTTGCCCGCCGCTAG<br>GACCTTTAAAACCCTTTGTAGTAGC<br>AGTATCTTCAGTTAAAACAAAATTA<br>TTAAAACTTTCAACAACGGATCTCT<br>TGGTTCTGGCATCGATGAAGAACGC<br>AGCGAAATGCGATAAGTAGTGTGA<br>ATTGCAGAATTCAGTGAATCATCGA<br>ATCTTTGAACGCACATTGCGCCCTT<br>CGGTATTCCGTTGGGCATGCCTGTTC<br>GAGCGTCATTTAAACCTTCAAGCTC<br>TGCTTGGTGTTGGGTGTTTGTTCCGC<br>CTAGTGCGTGGACTCGCCTTAAATT<br>CATTGGCAGCCGGTAAGTTGGCTTC<br>GTGCGCAGCACATTGTGTCGCGATC<br>CAGTTTACCTCCTTCCATCAAGCCTC<br>TTTTTTACTTTGACCTCGGATCAGGT<br>AGGGATACCCGCTGAACTTAA	GTGGTTCGTAAATCCAAAGGCTTTT<br>CCTGGGGACCGGCTGGAGTGTCCAC<br>GGCACTTTTTACAGGTGTGGTTATG<br>GCCGATGTCATTAGGAGAGCACGC<br>CCTCTACGAAAGGCGAAGTACGTCT<br>GCATGGAAGGTGCCGACAAATTAG<br>TGAGTCTAGACAATCAACAGAAAT<br>CATCATAGCTGACTTGAGGTAGCCG<br>AATGGATACTATGGGACCTCTGTTA<br>AGCTGAACTGGGTCATGGACCCCA<br>ATCGAGGAATCATGCTCGCCCACA<br>AAATGAACGGAGAAGCTCTAACAC<br>CTGATCATGGTAAACCCTTACGGGC<br>GGTAATTCCAGGTCAAATTGGCGGC<br>CGAAGTGTGAAGTGGCTTAAGAAG<br>CTTATTCTAACCGCGGAGCCGAGTG<br>ATAACTGGTACCACATTTACGACAA<br>CAGGGTACTCCCGTGAGTTATCTTTT<br>TGTCACTTTTGAGGCCTGAAACTAA<br>CATGGACAGAACGATGGTGGATCC<br>AGATGAGTCAGCTAGAAATCCGGC<br>ATGGTGGAAGGACGAGCGTTATGC<br>AATCTACGATTTAAACCCGAATGCC<br>GCAATTGCCCAACCAGCCCACGAT<br>GAACAGCTCCTCCTTGAAAATGCCC<br>CAGAGATGTACACAGCAAGAGGTT<br>ATGCATACAGCGGAGGTGGCCGCC<br>GCATCACACGAGCCGAAGTGTCAA<br>TTGACAAAGGCAAAACATGGAGAC<br>TGGCCAACATCGAATATGCCGAAG<br>ATCGCTATCGAGACTATGAAGACA<br>AACAACTGTTTGGCGGAAGACTAG<br>ACATGGACTGGCGGGAAACGTCCTT<br>CTGCTGGTGCTTTTGGAGCCTCGAC<br>ATACCAATGGTAGAACTCAAGGAC

			TCCAAGGATCTGTTGGTCCGTGCGA<br>TGGACGAGAGCATGAACATCATGC<br>CTAGAGACATGTATTGGTCTGTTCT<br>G
Dactylella cylindrospora	CBS325.70	AAGTCGTAACAAGGTCTCCGTTGGT<br>GAACCAGCGGAGGGATCATTACCG<br>AGTTTACAACTCCCAAACCCCTGTG<br>AACATACCTATTCTTGCTTCGGCGG<br>ACCACCCTCGCCTGACCGCGGGGG<br>CCCGCCAGAGGACCCAAAACCCAA<br>CTTTGTCTTTGTCTTGCAAACGAACT<br>TCTGAGTGGATTTTATAAATCAAAT<br>CAAAACTTTCAACAACGGATCTCTT<br>GGCTCTGGCATCGATGAAGAACGC<br>AGCGAAATGCGATAAGTAATGTGA<br>ATTGCAGAATTCAGTGAATCATCGA<br>ATCTTTGAACGCACATTGCGCCCGC<br>CAGTATTCTGGCGGGCATGCCTGTT<br>CGAGCGTCATTAACACCCCTCAAGC<br>CCCCGGGCTTGGTGTTGGGGATCGG<br>GAATCGCGCTGGCGGGATGGCCCCT<br>CGGGGTCCCCCCCGCCCGCCGCGCC<br>CGTCCCCCAAACGCATCGGCGGTCA<br>CGCCGCGGCCTCTTGCGCGTAGTAG<br>CTAACACCTCGCGCCGGAGCCCGTC<br>GTGGTCCACGCCGTAAAACCCCCG<br>ACTTTCTCAAAGTTGACCTCGAATC<br>AGGTAGGACTACCCGCTGAACTTAA	ATGGTGAGGAAAACTAAGGGGTTT<br>AGTTGGGGTGCGGCTGGTGTTAGTA<br>CTGCACTGTGGACGGGACCTATGCT<br>TAAGGATATAATCAAGCGAGCCAA<br>ACCGTTGAGGAGAGCGAAATATGT<br>ATGCATGGAAGGGGCCGACAAACT<br>ACCTAATGGGTACTATGGCACCAAT<br>GTTAGGCTGAGCTGGGTTAATGATC<br>CGAATAAGGGTATCATGCTAGCGC<br>ACAAGATGAACGGAGAAACGTTGA<br>CGCCCGACCATGGAAAGCCTATTCG<br>TGTTGTTATTCCAGGGCAAATCGGA<br>GGAAGGAGCGTCAAGTGGTTGACA<br>AAACTAATATTGACGGAAGGGCCG<br>AGTGAAAATTGGTACCACATCTATG<br>ATAATCGGGTATTACCGTAGGTACC<br>GACCGCCAGAAATTCTGTCTGGATT<br>TCTTACTAAACAATATATAGGACAA<br>TGGTTACCCCAGAAATGTCAACCCA<br>GGATCCAAGCTGGTGGACAGATGA<br>AAGATACGCTATCTACGACCTGTCT<br>ACAAACTCTGCGATCGCCTACCCGC<br>AACATGGTGAAACATTAAATCTAGC<br>GACCGCCCCAAAGACATATCGAGC<br>TCGAGGTTACGCTTACGGTGGAGGT<br>GGCAGACGAATCTCACGTGTTGAG<br>GTTTCAACCGACCGTGGGAATACAT<br>GGTCCCTAGCTAACATATCTTACCC<br>CGAGGACTTGTACCGGGATTATGAA<br>GGTACTTACCTTTTCGGTGGGAAGC<br>TCGATATGTACTGGCGAGATGCTTG<br>CCATTGTTGGTGCTTCTGGTCTATCG<br>ATCTCGACGTCGCTACGCTAGCGAG<br>CTCGGAGGGAATTCTGCTACGAGCT<br>ATGGACGAGTCGATGAACCTGCAG<br>CCGAGAGATATGTACTGGTCAGTAC<br>TT
Margaritispora aquatica	NNIBRFG339	AAGTCGTAACAAGGTTTCCGTAGGT<br>GAACCTGCGGAAGGATCATTACAG<br>AGTTCATGCCTCACGGGTAGATCTC<br>CCACCCTTGAATATTATACCTTAGTT<br>GCTTTGGTAGGCCGTGGAAACACTA<br>CGGGCTCCGGCTCGTACGTGCCTAC<br>CGAAGGAAACAAACTCTGTTTTTAG<br>TGATGTCTGAGTACTATAATAATAG<br>TTAAAACTTTCAACAACGGATCTCT<br>TGGTTCTGGCATCGATGAAGAACGC<br>AGCGAAATGCGATAAGTAATGTGA<br>ATTGCAGAATTCAGTGAATCATCGA<br>ATCTTTGAACGCACATTGCGCCCCG<br>TGGTATTCCGCGGGGCATGCCTGTT<br>CGAGCGTCATTACAACCCTCAAGCT<br>CACGCTTGGTATTGGAGCATGCGGT<br>TTCGCAGCCCTAAACTCAGTGGCGG<br>TGCCATCGAGCTCTGAGCGTAGTAA<br>ATTTTCTCGCTATAGGGTCTCGGTGG	CAAGTAAGAAAGTCGAAGGGATTC<br>TCGTGGGGAGCCGCTGGTGTGTCGA<br>CAGCGTTGTGGACCGGTGTCGCAAT<br>AAGCGAGCTGCTCAAGAGGGCCGT<br>CCCGCTTCGAGGAGCAAAGTACAT<br>GTGCATGGAAGGAGCCGACAAACT<br>ACCCAATGGTTATTATGGAACGAGT<br>ATCAAGTTGAACTGGGCAATGGAC<br>CCAAATAGGGGCATCATGTTGGCTC<br>ACAAGATGAACGGTGAGACTCTTA<br>CACCAGATCACGGCAAGCCGTTGC<br>GAGTGGTTATCCCCGGCCAGATTGG<br>AGGCCGAAGTGTGAAATGGCTCAA<br>GAGACTCATCGTCACAGCCGCACC<br>AAGTGATAACTGGTACCACATCTAC<br>GACAACAGAGTCCTTCCTACCATGG<br>TTACGCCTGAGATGGGTGCCGAGGA<br>TCCATCCTGGTGGACTGATGAGCGA<br>TACGCCATCTACGACCTGAGCACCA

		TTGCTTGCCAACAACCCCCCATTTT<br>ATCAGGTTGACCTCGGATCAGGTAG<br>GGATACCCGCTGAACTTAA	ACAGTGCAACAGCCTACCCAGCAC<br>ATGACGAGCAACTATGCCTCGTCGG<br>TGGACCAAAGACGTACAAGGTACG<br>AGGGTACGCCTACGGAGGTGGTGG<br>ACGTCGGGTCACTCGCGTCGAAGTC<br>ACTCTCGACAAGGGCAAATCCTGG<br>GCTTTAGCGAACATCAGATACCACG<br>AGGATGACTACCGGGAGGCAGCCG<br>AAGACGAGATGCTGTTCGGTGGTAA<br>GCTGGATATGGGATGGAGAGAGAC<br>CTGCTTTGCGTGGTGCTTCTGGGATA<br>TTGATTTGCCTGTGTCTGATCTCGCG<br>GTAGCTGGAGATGTCATGGTCCGAG<br>CGATGGACGAGAGCATGAATGTCC<br>AGCCACGTGATATGTACTGGAGTGT<br>CCTG
Thelonectria rubi	CBS 177.27	AAGTCGTAACAAGGTCTCCGTTGGT<br>GAACCAGCGGAGGGATCATTACCG<br>AGTTTACAACTCCCAAACCCCTGTG<br>AACATACCTATTCTTGCTTCGGCGG<br>ACCACCCTCGCCTGACCGCGGGGG<br>CCCGCCAGAGGACCCAAAACCCAA<br>CTTTGTCTTTGTCTTGCAAACGAACT<br>TCTGAGTGGATTTTATAAATCAAAT<br>CAAAACTTTCAACAACGGATCTCTT<br>GGCTCTGGCATCGATGAAGAACGC<br>AGCGAAATGCGATAAGTAATGTGA<br>ATTGCAGAATTCAGTGAATCATCGA<br>ATCTTTGAACGCACATTGCGCCCGC<br>CAGTATTCTGGCGGGCATGCCTGTT<br>CGAGCGTCATTAACACCCCTCAAGC<br>CCCCGGGCTTGGTGTTGGGGATCGG<br>GAATCGCGCTGGCGGGATGGCCCCT<br>CGGGGTCCCCCCCGCCCGCCGCGCC<br>CGTCCCCCAAACGCATCGGCGGTCA<br>CGCCGCGGCCTCTTGCGCGTAGTAG<br>CTAACACCTCGCGCCGGAGCCCGTC<br>GTGGTCCACGCCGTAAAACCCCCG<br>ACTTTCTCAAAGTTGACCTCGAATC<br>AGGTAGGACTACCCGCTGAACTTAA	ATGGTGCGAAAAACAAAGGGCTTC<br>TCATGGGGAGCAGCGGGCTTGTCTA<br>CGGCGCTGTGGACTGGTGTGCCAAT<br>CCACAACCTTCTCGCGACTGCACGA<br>CCAAAGCGAGGTGCGAGATACGTC<br>TGCTTCGAGGGTGCCGACAAGCTTC<br>CCAACGGTTACTATGGGACATCCAT<br>CAAGCTGAACTGGTGCATGGATCCC<br>AACAGAGGCGTCATGGTCGCCCATC<br>GGATGAACGGAGAGAAGTTGCACC<br>CCGATCATGGGAAGCCCGTTCGAAT<br>CATCATCCCCGGCCAAATCGGCGG<br>AAGAAGCGTCAAGTGGCTGAAGAG<br>AATCATCGTCACCCCTGGGCCGAGC<br>GACAATTGGTACCACATCTTTGACA<br>ACCGCGTGTTGCCTACCATGATCAC<br>CCCTGAAGCCAGCGCCGACTTGCCC<br>GACGTGTGGAGGGATGAGAAGTAC<br>GCCATCTACGATCTCAACGTCAATA<br>GCGCCATATGCTTCCCTGGCCACCA<br>AGAAACGCTACCCCTGTCCGATGGT<br>CCGGCCAGTTATACGGCGCGAGGA<br>TACGCATACAGCGGTGGTGGGAGA<br>CGAGTCACAAGGGTAGAGGTCACA<br>CTGGACAAGGGCAAGTCATGGAGA<br>CTCGCAAAAATAAACTACCCCGAA<br>GACGAGTACCGACAAGCTCCAGAC<br>GACGACAGCCTGTACGGCGGGACG<br>GTGGACGTGTGGTGGCGAGAGACG<br>TGTTTCTGTTGGTGTTTTTGGGAGCT<br>CGACATAGCGCTCGACGACATCAG<br>GGGGGCCGACGACATCATGATTCG<br>CGCAATGGACGAGTCCATGATGGTC<br>CAGCCTCGCGACATGTACTGGAGCG<br>TCTTG

Table S2. Number of clone colonies used for enzymatic restriction (EcoRI and BamHI), DNA inserts sent for sequencing and sequences utilized in the alignments of each fungal isolate (using all the sequencing efforts; both digestions with EcoRI and BamHI). nt, not tested.

Aquatic hyphomycete	Strain code	Number of			
		Clone colonies		Inserts sequenced	Sequences used
		EcoRI	BamHI		
Alatospora acuminata	UMB-741.11	8	Nt	4	4
Alatospora pulchella	UMB-1115	8	Nt	6	5
Anguillospora crassa	UMB-1150	8	Nt	5	4
Anguillospora filiformis	UMB-225.02	7	6	5	1 (+1 partial)
Anguillospora filiformis	UMB-232.02	8	4	6	1 (+1 partial)
Articulospora tetracladia	UMB-712.10	6	5	7	1 (+ 2 partials)
Articulospora tetracladia	UMB-719.10	8	Nt	6	3
Collembolispora barbata	UMB-88.01	7	5	4	1 (+1 partial)
Dimorphosphora foliicola	UMB-1119	8	Nt	5	4
Lemmoniera aquatica	UMB-594.10	8	3	7	3
Tetracladium apiense	UMB-535.10	10	Nt	7	7
Tetracladium marchalianum	UMB-1028.13	8	Nt	5	3
Tetracladium marchalianum	UMB-1079.13	6	Nt	6	6
Tricladium chaetocladium	UMB-904.12	8	Nt	8	6
Tricladium chaetocladium	UMB-1116	10	1	4	3
Tricladium splendens	UMB-100.01	8	7	7	1
Tricladium splendens	UMB-1117	8	Nt	5	4
Varicosporium elodeae	UMB-713.10	8	5	4	1

Table S3. Evolutionary divergence (%) between aquatic hyphomycete species (interspecific level) based on ITS-5.8S-ITS2 sequences. The number of base substitutions per site from averaging overall sequence pairs and single isolates between the species is shown. Analyses were performed resorting to the maximum composite likelihood-parameter method. The lower half of the matrix contains p-distance values (in percentage) and the upper half values correspond to the standard error for each p-distance.

Species	1	2	3	4	5	6	7	8	9	10	11	12	13	14	15	16	17	18	19	20	21
Alatospora acuminata (1)		0.9%	1.6%	1.6%	1.8%	1.5%	1.7%	1.5%	1.6%	1.7%	1.5%	1.5%	1.7%	1.7%	1.7%	1.7%	1.7%	1.6%	1.5%	1.6%	1.6%
Alatospora pulchella (2)	**4.4%**		1.7%	1.6%	1.8%	1.4%	1.7%	1.5%	1.7%	1.6%	1.6%	1.5%	1.6%	1.6%	1.6%	1.7%	1.7%	1.7%	1.6%	1.7%	1.5%
Anguillospora crassa (3)	**19.3%**	**19.0%**		1.6%	1.7%	1.5%	1.9%	1.5%	1.9%	1.5%	1.7%	1.6%	1.7%	1.7%	1.6%	1.7%	1.8%	1.9%	1.5%	0.6%	1.6%
Anguillospora filiformis (4)	**17.1%**	**17.4%**	**16.9%**		1.8%	1.0%	1.9%	1.5%	1.9%	1.6%	1.1%	1.1%	1.6%	1.6%	1.6%	1.6%	1.6%	1.9%	1.5%	1.6%	1.0%
Aquanectria penicillioides (5)	**21.6%**	**21.7%**	**23.9%**	**21.1%**		1.7%	1.8%	1.7%	1.5%	1.7%	1.7%	1.7%	1.7%	1.7%	1.7%	1.6%	1.7%	1.5%	1.7%	1.7%	1.8%
Articulospora tetracladia (6)	**16.0%**	**14.7%**	**16.1%**	**6.6%**	**21.2%**		1.7%	1.4%	1.7%	1.6%	1.0%	0.9%	1.4%	1.4%	1.4%	1.5%	1.5%	1.7%	1.4%	1.5%	0.8%
Clavariopsis aquatica (7)	**21.4%**	**21.9%**	**24.0%**	**22.7%**	**25.8%**	**19.2%**		1.8%	1.8%	1.8%	1.7%	1.7%	1.7%	1.8%	1.8%	1.8%	1.8%	1.8%	1.7%	1.8%	1.8%
Collembolispora barbata (8)	**16.4%**	**15.9%**	**17.9%**	**15.7%**	**24.2%**	**14.1%**	**23.0%**		1.7%	1.6%	1.6%	1.5%	1.6%	1.7%	1.6%	1.6%	1.7%	1.7%	1.5%	1.5%	1.4%
Dactylella cylindrospora (9)	**21.9%**	**21.3%**	**24.0%**	**23.5%**	**14.3%**	**22.5%**	**26.3%**	**22.3%**		1.8%	1.8%	1.7%	1.7%	1.8%	1.7%	1.7%	1.8%	0.0%	1.7%	1.8%	1.8%
Dimorphospora foliicola (10)	**20.2%**	**19.2%**	**16.7%**	**16.0%**	**22.0%**	**16.3%**	**24.4%**	**21.8%**	**23.7%**		1.6%	1.6%	1.6%	1.6%	1.6%	1.6%	1.6%	1.8%	1.5%	1.5%	1.6%
Lemonniera aquatica (11)	**17.2%**	**16.7%**	**18.4%**	**8.1%**	**20.9%**	**6.1%**	**20.4%**	**16.7%**	**23.2%**	**17.4%**		0.5%	1.4%	1.5%	1.5%	1.5%	1.5%	1.8%	1.4%	1.6%	1.1%
Margaritispora aquatica (12)	**16.2%**	**15.3%**	**17.2%**	**7.3%**	**21.0%**	**4.7%**	**20.4%**	**15.5%**	**22.2%**	**16.6%**	**1.5%**		1.4%	1.5%	1.4%	1.5%	1.5%	1.7%	1.3%	1.6%	1.0%
Tetracladium apiense (13)	**18.1%**	**17.5%**	**18.7%**	**16.8%**	**23.1%**	**13.9%**	**19.9%**	**16.6%**	**21.5%**	**19.4%**	**13.9%**	**13.2%**		0.7%	0.6%	0.7%	0.7%	1.7%	1.5%	1.7%	1.5%
Tetracladium furcatum (14)	**18.7%**	**18.1%**	**18.7%**	**16.1%**	**22.4%**	**14.0%**	**21.5%**	**17.2%**	**21.6%**	**18.3%**	**14.8%**	**13.9%**	**2.5%**		0.7%	0.5%	0.0%	1.8%	1.6%	1.7%	1.5%
Tetracladium marchalianum (15)	**18.3%**	**17.5%**	**18.8%**	**17.6%**	**23.1%**	**14.3%**	**21.0%**	**17.6%**	**21.3%**	**18.5%**	**14.5%**	**14.1%**	**2.0%**	**3.0%**		0.8%	0.7%	1.7%	1.5%	1.6%	1.5%
Tetracladium maxilliforme (16)	**18.8%**	**18.2%**	**18.0%**	**16.2%**	**22.8%**	**14.7%**	**21.0%**	**17.3%**	**21.9%**	**18.4%**	**15.3%**	**14.4%**	**2.9%**	**1.4%**	**3.6%**		0.5%	1.7%	1.6%	1.7%	1.5%
Tetracladium setigerum (17)	**19.0%**	**18.4%**	**18.8%**	**16.4%**	**22.6%**	**14.3%**	**21.6%**	**17.5%**	**21.7%**	**18.6%**	**15.1%**	**14.2%**	**2.5%**	**0.0%**	**3.0%**	**1.4%**		1.8%	1.6%	1.7%	1.5%
Thelonectria rubi (18)	**21.9%**	**21.3%**	**24.0%**	**23.5%**	**14.3%**	**22.5%**	**26.3%**	**22.3%**	**0.0%**	**23.7%**	**23.2%**	**22.2%**	**21.5%**	**21.6%**	**21.3%**	**21.9%**	**21.7%**		1.7%	1.8%	1.8%
Tricladium chaetocladium (19)	**17.8%**	**18.0%**	**16.8%**	**17.1%**	**20.4%**	**15.8%**	**23.2%**	**20.1%**	**21.6%**	**15.2%**	**16.2%**	**15.0%**	**17.5%**	**18.3%**	**17.0%**	**18.0%**	**18.6%**	**21.6%**		1.4%	1.5%
Tricladium splendens (20)	**20.1%**	**20.0%**	**2.5%**	**16.5%**	**23.3%**	**16.0%**	**23.1%**	**18.2%**	**23.4%**	**15.8%**	**17.5%**	**16.7%**	**18.4%**	**18.9%**	**18.0%**	**18.1%**	**18.9%**	**23.4%**	**16.4%**		1.6%
Varicosporium elodeae (21)	**16.7%**	**15.7%**	**17.4%**	**5.7%**	**21.6%**	**3.8%**	**21.5%**	**14.4%**	**23.2%**	**15.8%**	**6.8%**	**5.7%**	**14.4%**	**14.5%**	**14.2%**	**14.9%**	**14.7%**	**23.2%**	**17.1%**	**16.9%**	

Table S4. Evolutionary divergence (%) within aquatic hyphomycete species (intraspecific level) based on ITS-5.8S-ITS2 and Nitrate reductase sequences. The number of base substitutions per site from averaging overall sequence pairs and single isolates between the species is shown. Analyses were performed resorting to the maximum composite likelihood-parameter method. Values on the left side correspond to p-distances (in percentage) and values on the right side correspond to the standard error (SE) for each p-distance.

Species	ITS-5.8S-ITS2		Nitrate reductase	
	Divergence (%)	SE	Divergence (%)	SE
Anguillospora filiformis	**0.0%**	0.0%	**0.0%**	0.0%
Articulospora tetracladia	**0.2%**	0.2%	**2.1%**	0.5%
Tetracladium marchalianum	**0.2%**	0.2%	**0.7%**	0.2%
Tricladium chaetocladium	**0.0%**	0.0%	**30.0%**	1.5%
Tricladium splendens	**0.0%**	0.0%	**0.0%**	0.0%

Table S5. Evolutionary divergence (%) between aquatic hyphomycete species (interspecific level) based on nitrate reductase partial nucleotide sequences. The number of base substitutions per site from averaging overall sequence pairs and single isolates between the species is shown. Analyses were performed resorting to the maximum composite likelihood-parameter method. The lower half of the matrix contains p-distance values (in percentage) and the upper half values correspond to the standard error for each p-distance.

Species	1	2	3	4	5	6	7	8	9	10	11	12	13	14	15	16	17	18	19	20	21
Alatospora acuminata (1)		1.2%	1.5%	1.5%	1.6%	1.5%	1.6%	1.5%	1.6%	1.5%	1.5%	1.5%	1.5%	1.5%	1.5%	1.6%	1.5%	1.5%	1.2%	1.5%	1.5%
Alatospora pulchella (2)	**13.3%**		1.5%	1.4%	1.6%	1.5%	1.7%	1.5%	1.6%	1.5%	1.5%	1.4%	1.5%	1.5%	1.5%	1.5%	1.5%	1.6%	1.3%	1.4%	1.5%
Anguillospora crassa (3)	**26.2%**	**28.0%**		1.4%	1.6%	1.5%	1.5%	1.4%	1.5%	1.4%	1.4%	1.5%	1.4%	1.4%	1.5%	1.4%	1.5%	1.6%	1.2%	0.9%	1.4%
Anguillospora filiformis (4)	**29.2%**	**29.8%**	**21.9%**		1.5%	1.3%	1.6%	1.5%	1.6%	1.4%	1.3%	1.3%	1.5%	1.5%	1.5%	1.4%	1.5%	1.5%	1.2%	1.4%	1.3%
Aquanectria penicillioides (5)	**37.4%**	**36.9%**	**34.9%**	**34.5%**		1.5%	1.6%	1.6%	1.6%	1.6%	1.6%	1.5%	1.6%	1.6%	1.6%	1.6%	1.5%	1.4%	1.2%	1.6%	1.5%
Articulospora tetracladia (6)	**30.2%**	**30.1%**	**23.9%**	**19.7%**	**33.6%**		1.6%	1.4%	1.5%	1.5%	1.3%	1.3%	1.4%	1.5%	1.4%	1.5%	1.5%	1.5%	1.1%	1.4%	1.3%
Clavariopsis aquatica (7)	**33.7%**	**32.6%**	**32.0%**	**33.3%**	**36.6%**	**32.1%**		1.6%	1.4%	1.6%	1.6%	1.6%	1.6%	1.7%	1.7%	1.7%	1.6%	1.6%	1.4%	1.6%	1.6%
Collembolispora barbata (8)	**31.2%**	**32.1%**	**24.1%**	**26.3%**	**35.7%**	**24.9%**	**33.1%**		1.6%	1.5%	1.4%	1.4%	0.0%	1.0%	1.0%	1.0%	1.1%	1.4%	1.1%	1.4%	1.4%
Dactylella cylindrospora (9)	**32.9%**	**34.5%**	**33.1%**	**34.5%**	**39.0%**	**32.3%**	**37.1%**	**36.8%**		1.5%	1.5%	1.5%	1.6%	1.5%	1.6%	1.6%	1.6%	1.6%	1.3%	1.5%	1.3%
Dimorphospora foliicola (10)	**28.7%**	**29.9%**	**22.1%**	**25.4%**	**35.6%**	**25.8%**	**31.8%**	**24.7%**	**36.1%**		1.5%	1.6%	1.5%	1.4%	1.4%	1.4%	1.3%	1.5%	1.1%	1.4%	1.5%
Lemonniera aquatica (11)	**30.4%**	**31.0%**	**22.9%**	**22.4%**	**34.6%**	**17.5%**	**32.6%**	**24.8%**	**33.8%**	**26.1%**		0.7%	1.4%	1.5%	1.5%	1.5%	1.5%	1.6%	1.1%	1.4%	1.3%
Margaritispora aquatica (12)	**29.3%**	**29.9%**	**23.3%**	**22.4%**	**33.6%**	**17.9%**	**32.2%**	**24.6%**	**34.1%**	**25.7%**	**4.8%**		1.4%	1.5%	1.5%	1.5%	1.5%	1.6%	1.1%	1.5%	1.2%
Tetracladium apiense (13)	**31.2%**	**32.1%**	**24.1%**	**26.3%**	**35.7%**	**24.9%**	**33.1%**	**0.0%**	**36.8%**	**24.7%**	**24.8%**	**24.6%**		1.0%	1.0%	1.0%	1.1%	1.4%	1.1%	1.4%	1.4%
Tetracladium furcatum (14)	**30.0%**	**32.0%**	**23.5%**	**26.2%**	**36.0%**	**25.4%**	**32.3%**	**10.6%**	**37.0%**	**23.8%**	**24.6%**	**24.4%**	**10.6%**		1.0%	1.0%	1.0%	1.5%	1.2%	1.4%	1.5%
Tetracladium marchalianum (15)	**29.8%**	**32.2%**	**25.1%**	**26.6%**	**35.4%**	**25.8%**	**32.8%**	**11.5%**	**37.2%**	**22.8%**	**26.3%**	**25.5%**	**11.5%**	**11.3%**		1.0%	1.0%	1.4%	1.1%	1.5%	1.5%
Tetracladium maxilliforme (16)	**30.8%**	**31.8%**	**24.6%**	**26.3%**	**35.6%**	**26.2%**	**33.3%**	**10.1%**	**37.8%**	**24.2%**	**26.4%**	**26.0%**	**10.1%**	**8.9%**	**10.5%**		0.9%	1.4%	1.2%	1.4%	1.4%
Tetracladium setigerum (17)	**31.8%**	**32.3%**	**25.1%**	**27.0%**	**34.9%**	**27.0%**	**32.3%**	**12.3%**	**38.4%**	**24.3%**	**27.4%**	**26.7%**	**12.3%**	**9.7%**	**12.3%**	**8.2%**		1.4%	1.2%	1.4%	1.4%
Thelonectria rubi (18)	**34.8%**	**34.6%**	**32.0%**	**32.1%**	**22.2%**	**32.4%**	**34.7%**	**34.9%**	**37.9%**	**33.2%**	**34.0%**	**32.6%**	**34.9%**	**35.0%**	**34.5%**	**34.4%**	**32.7%**		1.2%	1.5%	1.6%
Tricladium chaetocladium (19)	**29.8%**	**30.1%**	**26.6%**	**25.9%**	**35.4%**	**25.3%**	**33.8%**	**27.5%**	**37.6%**	**26.6%**	**26.4%**	**26.5%**	**27.5%**	**27.8%**	**26.5%**	**28.1%**	**27.1%**	**34.3%**		1.2%	1.1%
Tricladium splendens (20)	**26.7%**	**28.2%**	**7.4%**	**21.6%**	**35.1%**	**24.8%**	**32.5%**	**24.6%**	**32.9%**	**21.7%**	**23.9%**	**24.5%**	**24.6%**	**23.6%**	**23.7%**	**24.7%**	**25.7%**	**33.1%**	**26.5%**		1.3%
Varicosporium elodeae (21)	**29.0%**	**30.7%**	**22.7%**	**21.5%**	**34.8%**	**16.6%**	**31.1%**	**25.4%**	**33.8%**	**25.2%**	**17.0%**	**16.7%**	**25.4%**	**25.4%**	**25.7%**	**26.0%**	**26.3%**	**33.5%**	**26.7%**	**23.0%**	
